# Setting the research agenda for Council of Southern Africa Football Associations (COSAFA) women’s football: stakeholder perspectives

**DOI:** 10.1186/s13102-026-01692-y

**Published:** 2026-04-17

**Authors:** Fidelis Chibhabha, Fatou Binetou Ba Ndiaye, Menzi Obed Ngcobo, Nonhlanhla Sharon Mkumbuzi

**Affiliations:** 1 Lancaster Medical School, Lancaster University, Lancaster, LA1 4AT UK; 2 Fédération Internationale de Football Association (FIFA), Member Associations Division, Paris, France; 3 Hollywoodbets (PTY) Ltd, Durban, South Africa; 4 Department of Philosophy, Durham University, Durham, DH1 3HN UK; 5 Department of Human Movement Science, Nelson Mandela University, Qheberha, South Africa; 6 Department of Rehabilitation, Midlands State University, Gweru, Zimbabwe; 7 NtombiSport (PTY) Ltd, Cape Town, South Africa

**Keywords:** Research agenda, Africa, Stakeholder perspectives, Football medicine research, Women’s football

## Abstract

Despite the increasing global interest in women’s football research, relatively less attention has been devoted to African women’s football. Where research is conducted, the agenda tends to be driven by researchers usually without input from football stakeholders. Hence, research priorities may not reflect the needs of football stakeholders or address the real problems they face. Utilising the stakeholder theory framework, this cross-sectional study established self-reported medical and performance research priorities of stakeholders in women’s football in the Council of Southern Africa Football Associations (COSAFA) region. A self-administered questionnaire ranking 17 areas of research was administered to women football players (≥18 years old), and their support personnel (e.g., coaches, medical personnel, administrators) participating in the 2022 TOTAL Energies CAF Women’s Champions League (CAFWCL) COSAFA Qualifiers and 2022 COSAFA Women’s Championship. One hundred and forty-one (*n* = 141) respondents from 10 countries participated. The top five research areas considered to be highly relevant to women’s football in the COSAFA region were: (1) developing youth players (*n* = 115; 82%) (2), optimising physical conditioning (*n* = 112; 79%) (3), understanding female related health issues (*n* = 107; 76%) (4), improving technical skills (*n* = 106; 75%) (5), how to come back stronger after injury (*n* = 106;75%). Given a hypothetical USD $1 million research fund, stakeholders would prioritise (1) development of youth players (*n* = 70; 17%) (2), improvement of technical skills (*n* = 68; 16%), and (3) injury prevention (*n* = 33; 8%) as their top three areas for research focus. Identifying stakeholders’ research priorities is important as this can inform research directions and funding priorities in African women’s football.

**Supplementary Information:**

The online version contains supplementary material available at 10.1186/s13102-026-01692-y.

## Background

The increase in global popularity of women’s football in recent years has been accompanied by the development of the game in Africa [1, 2]. Currently, several African countries have domestic women’s leagues and national teams that play in regional and international tournaments organised under the Confederation of African Football (CAF) or Fédération Internationale de Football Association (FIFA). In 2023, four African countries Nigeria, South Africa, Morocco, and Zambia qualified and participated in the FIFA Women’s World Cup 2023™ with Nigeria reaching the Round of 16 [3]. In the 2023 FIFA Women’s Football Member Association Survey Report, 16.6 million women and girls are currently playing organised football worldwide which represents a 24% increase from the number recorded in 2019. Of these women and girls, around 1.75 million are in the Confederation of African Football (CAF) region [4].

The current and projected increase in participation and the increased drive towards professionalization of women’s football has led to increased interest in the general discourse, and women’s football is attracting more attention from researchers, though not yet to the same extent as men’s football [5, 6]. Even less attention is devoted to women’s football research in low- and middle-income settings, such as in Africa, which continue to be on the margins of methodological, theoretical, and empirical discussion [7]. To the authors’ knowledge, there are presently no reviews that audit recent studies on African women’s sports. However, existing research on African women’s football is dominated by small cross-sectional surveys focusing on areas such as injury surveillance, the menstrual cycle, and sexual health knowledge among others [8–12]. Due to the inherent limitation of cross-sectional studies to establish causal inferences, they are usually insufficient to inform or change practice on their own [13]. Further, global trends show that Africa accounts for 1% of research publications in sports medicine [14]. This low research output reflects the low funding in general research that is ubiquitous in most African countries. Notably, Africa produces only 2% of the world’s overall research output and accounts for 1% of global investments in research and development [15]. In such a setting of resource scarcity, it is the women, children, and those with disabilities who bear the brunt of these shortages and sports research is no different. This low investment in research and the ensuing relative invisibility in scientific literature means that issues that pertain to African women’s football are generally not being investigated as a matter of course. Consequently, stakeholders in African women’s football rely on information that is derived from male studies or women and girls from high income countries in the Global North, which may not be directly applicable to their circumstances [7]. Hence, more research needs to be conducted in African women’s football to better inform policy, budgets, rules, and training plans.

In order for this proposed research in African women’s football to be impactful, it needs to align with the needs of stakeholders in African women’s football. Hence, this study adopted the stakeholder theory perspective to better understand specific areas which stakeholders of African women’s football in the Council of Southern Africa Football Associations (COSAFA) region consider as research priorities. Originally developed in management studies to help organizations to be cognizant of stakeholder input to achieve superior performance [16], the stakeholder theory has since been applied to various disciplines. In the sporting context, the theory recognises that maximum consideration of stakeholder interests is an essential element to the overall success of sporting organisations [17]. In our case, we engaged stakeholders of the African women’s football ecosystem, to understand what their needs are and use these to inform future and impactful research directions. This aligns with existing research in women sport which emphasises the need to rethink research through collective effort of those involved for the betterment of women sport [18].

To date, studies to identify stakeholder views on women’s sports have been carried out mostly in stakeholders from high income countries [18–20]. Consequently, research priorities and recommended areas for further research in women’s football identified in the current literature may differ and not be relevant to the needs of stakeholders in African women’s football. These nuances are important as they would guide optimal allocation of resources in women’s football research. In developing relevant and meaningful research priorities in sports and exercise medicine research, the ‘voice’ of the athletes or stakeholders is critical especially in resource limited settings as it helps to successfully address the most urgent, preventable, or important problems according to the needs and context of the stakeholders [21, 22]. Therefore, the aim of this study was to identify specific areas which stakeholders of African women’s football in the Southern African region consider as priorities in football medicine and performance research. These insights can directly inform policy makers and direct investment of research resources in areas that are relevant to the stakeholders.

## Methods

### Study design, participants, and setting

This was a descriptive, cross-sectional survey conducted in accordance with the Strengthening the Reporting of Observational Studies in Epidemiology (STROBE) guidelines [23] (Supplementary Information 1). The target population consisted of women football players (above 18 years old), and their support personnel; namely, coaches, medical doctors, physiotherapists, team managers, administrators, and referees, participating in the 2022 TOTAL Energies Confederation of African Football Women’s Champions League Qualifiers – COSAFA (CAFWCL) and the Council of Southern Africa Football Associations (COSAFA) Women’s Championship. The COSAFA Women’s Championship is an association football tournament of 10 to 12 national teams from the 14 member associations in the Southern African bloc (Angola, Botswana, Comoros Islands, Eswatini, Lesotho, Malawi, Mauritius, Mozambique, Namibia, Reunion, Seychelles, South Africa, Zambia, and Zimbabwe) and the CAF Women’s Champions league is a continental club competition of eight [8] women’s football clubs that are champions in their respective domestic club football leagues in each of CAF’s six regions. All athletes and athlete support personnel (above 18 years old) participating in African Women’s Football tournaments in 2022 were eligible to participate in the study if they could read and write in basic English, French, or Portuguese. Participants who were under 18 years of age or unable to read or write were excluded from the study.

### Data collection and ethical considerations

The players were requested to be present for the study at a time and day that was convenient for them during their team’s time in the relevant tournament. This pragmatic approach for recruitment and testing was adopted to minimise disruption of their training, competitive, and recovery schedules. At the visit, participants completed a self-administered online questionnaire (Supplementary Information 2), which is adapted from McCall et al., 2020 (unpublished). Section A of the questionnaire collected data on self-reported demographic data of respondents. In Section B participants were asked to rank topics they considered relevant for targeted medical research in African Women’s football from a list of seventeen topics. The ranking was based on a 4 point-Likert scale (Not at all Relevant, Somewhat Relevant, Relevant and Highly Relevant). In addition, they could add their own self-identified highly relevant areas of research in the open-ended part of Section B. In Section C, participants were asked to indicate top three research areas out of seventeen that they would invest a hypothetical USD$1 million. Additionally, they could identify any other areas not listed in the open-ended part of Section C.

Permission to conduct the studies at the tournaments was obtained from the tournament organisers and ethical approval was obtained from the Research and Ethics Review Committee of the Faculty of Medicine, Midlands State University, Zimbabwe (MSUFMEC 007/06/22). Participation in the study was voluntary, and it was conducted according to the principles of the Helsinki Declaration. Study participants were not included in the study unless they signed a consent form and only after the investigator had provided substantial verbal and written explanation of the study, including the risks involved. Furthermore, participants had the right to withdraw from the study without prejudice. No identifying data were collected from the questionnaire.

### Data analyses

Quantitative data were processed on 2021 Microsoft Excel Spreadsheets and SPSS^®^ (IBM, 30.0.0). Descriptive statistics of normally distributed data were presented as mean (± standard deviation) or as absolute numbers (n) or percentages (%). Where data were not normally distributed, they were presented as median [interquartile range]. The open-ended responses were thematically analysed using Braun and Clarke’s inductive approach [24]. To enhance the rigour of the process, FC and NSM independently contributed to the initial analysis of responses into central themes. Later, findings were shared with all authors and differences in interpretation were resolved through discussion.

## Results

### Demographic characteristics of the participants

Approximately 350 individuals (including players, coaches, medical doctors, physiotherapists, team managers, administrators, referees) from 10 COSAFA member associations participated in the 2022 COSAFA Women’s Championship and approximately 250 individuals participated in the 2022 CAFWCL COSAFA Qualifiers. Of the total 600 individuals from the two competitions, 141 (~ 23.5%) participants responded to the questionnaire. The median age of the participants at recruitment was 30 years (range 19–66 years). 70% (*n* = 98; 70%) of the study respondents were women, (*n* = 41; 29%) were men and (*n* = 2; 1%) preferred not to say. Most participants were football players (*n* = 56; 40%); the highest level of education attained by 40% (*n* = 56) of the respondents was secondary school level, 25% (*n* = 35) had a bachelor’s degree, while 1% (*n* = 2) had doctorates. Other professionals who participated in the study included coaches (*n* = 17; 12%), referees (*n* = 6; 4%), team medical doctors (*n* = 3; 2%), physiotherapists (*n* = 1; 1%), team managers (*n* = 5; 3.5%), and administrators (*n* = 19; 13.5%). More demographic data are presented in Table 1.

**Table 1:** Demographic characteristics of study participants (*n* = 141). The values are presented as absolute figures (*n*) and percentages (%)

Characteristic	Frequency(% )
Sex	
Female	98(70)
Male	41(29)
Prefer not to say	2(1)
Country of residence	
South Africa	76(54)
Zambia	4(3)
Zimbabwe	2(1)
Eswatini	17(12)
Lesotho	3(2)
Mauritius	3(2)
Botswana	8(6)
Tanzania	3(2)
Namibia	24(17)
Switzerland	1(1)
Highest educational qualification attained	
Doctorate	2(1)
Masters	10(7)
Bachelors	35(25)
Diploma	38(27)
Secondary school	56(40)
Level of football participation	
Amateur	35(25)
Semi-professional	27(19)
Professional	66(66)
Other	13(9)
Role in football	
Football player	56(40)
Administrator	19(13)
Coach	17(12)
Healthcare personnel (team doctor, PT, S&C coach)	6(4)
Team/welfare manager	5(4)
Match officials (e.g., referees)	6(4)
Other	32(23)
Country of football affiliation/club	
South Africa	75(53)
Namibia	22(16)
Botswana	6(4)
Eswatini	16(11)
Other**	22(16)

**Other – Zambia 4(3%), Lesotho 1(1%), Mauritius 3(2%), Turkey 1(1%), Germany 1(1%), and Tanzania 3(2%), not a club 8(6%)

### Research priorities

Out of 17 areas of research presented in the questionnaire, the participants identified their top five highly relevant areas for targeted research in African women’s football as: developing youth players (technical, tactical, mental, physical attributes) (82%), optimising physical conditioning (strength, speed, power, and endurance) (79%), understanding female related health issues (76%), how to come back stronger after injury (75%), and improving technical skills (75%) (Table 2).

**Table 2:** Research priorities for African women’s football as identified by stakeholders in African women’s football (*n* = 141)

Proposed area for targeted research	Highly relevant *n* (%)	Relevant *n* (%)	Somewhat Relevant *n* (%)	Not at all relevant *n* (%)
Improving technical skills	106(75)	27(19)	6(4)	2(1)
Enhancing tactical knowledge and application	95(67)	37(26)	6(4)	3(2)
Optimising physical conditioning (strength, speed, power, and endurance)	112(79)	18(13)	9(6)	2(1)
Anti-doping	76(54)	32(23)	25(18)	8(6)
Optimising diet and nutrition	94(67)	37(26)	9(6)	1(1)
Optimising recovery systems	98(70)	32(23)	9(6)	2(1)
Improving psychological attributes and skills	100(71)	30(21)	10(7)	1(1)
How to prevent injuries	103(73)	29(21)	7(5)	2(1)
How to come back stronger after injury	106(75)	29(21)	5(4)	1(1)
Developing youth players (technical, tactical, mental, physical)	115(82)	18(13)	7(5)	1(1)
Developing para/disability football	101(72)	26(18)	11(8)	3(2)
Understanding the impact of potential female health related factors (e.g. menstrual cycle, pregnancy, menopause)	107(76)	26(18)	7(5)	1(1)
Enhancing leadership and coaching behaviours	96(68)	35(25)	9(6)	1(1)
Developing equipment/technologies targeting high performance female athletes	96(68)	33(23)	11(8)	1(1)
Preparing for and transitioning into retirement (mental and physical health)	103(73)	25(18)	10(7)	3(2)
Transgender athletes and athletes with differences of sexual development	86(61)	30(21)	16(11)	9(6)
Investigating equity, diversity, and inclusion in football	96(68)	34(24)	11(8)	0(0)

The five areas that were ranked lowest for targeted research in African women’s football were: transgender athletes and athletes with variations of sexual development (6%), anti-doping (6%), developing para/disability football (2%), preparing for, and transitioning into retirement (2%), and enhancing tactical knowledge and application (2%)** (**Table 2).

In the open-ended section of the questionnaire, participants identified other areas of targeted research, which we classified into four themes that include development and support of women and girls’ football, player well-being, personal development and professionalism, and challenges in women’s football (Table 3).

**Table 3:** Themes covering other self-identified areas relevant to stakeholders for targeted research on African women football players (*n* = 141)

Theme area	Specific issues mentioned
Development and support of women’s football	• improving infrastructure and training facilities • improving sponsorship for women’s football • developing women’s football academies • increasing women involvement in football • identification of talented women and girls in rural areas • increase the number of women coaches
Player well-being	• fatigue related issues • emotional support systems for players • career support from home country
Personal and professional development	• introducing league awards for every game e.g., woman of the match • balancing education and sport • financial management training • discipline
Challenges in women’s football	• poor training facilities • lack of opportunities for girls in rural and public schools • problems associated with male coaches e.g. favouritism, communication and trust barriers

**Fig. 1:**
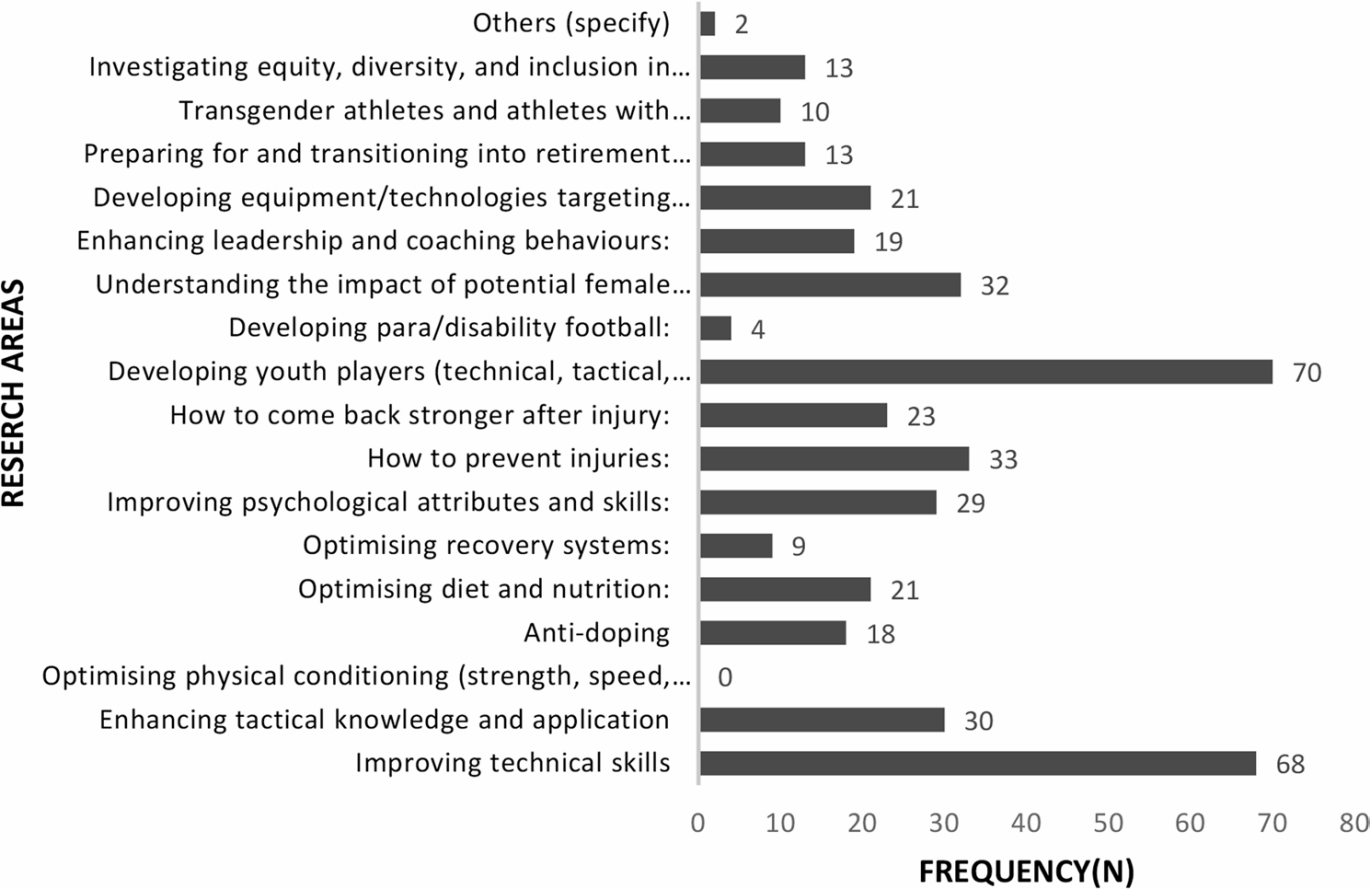
Research areas stakeholders would invest money to carry out research on African women football players (*n* = 141)

If given a hypothetical budget of USD1 million, the top three areas that the participants would choose to conduct research on in African women’s football were developing youth players (technical, tactical, mental, physical attributes) (51%), improving technical skills (48%), and how to prevent injuries (24%) **(**Fig. 1**).**

## Discussion

Drawing from the stakeholder theory [16], this study explored priority areas for research among stakeholders of women’s football in the Council of Southern Africa Football Associations (COSAFA) region. The findings showed that the top five areas that stakeholders considered highly relevant for targeted research on African women’s football were: developing youth players, optimising physical conditioning, understanding female related health issues, improving technical skills, and how to come back stronger after injury. Additionally, given a hypothetical budget of USD1 million, the top three areas they would invest in for research in African women’s football were: developing youth players, improving technical skills, and how to prevent injuries. These three areas can be described as ‘mature’ research themes [25], that have been extensively researched in countries where women’s football is advanced but are still under-researched in Africa.

The development of youth football players was ranked as highly relevant for targeted research in the present cohort. Additionally, in the open-ended sections, stakeholders suggested research involving talent identification, especially in rural players. This is similar to a programme carried out by The South African Football Association [26], but its impact is yet to be documented. The development of youth football players is important in nurturing talented young players (physically, technically, tactically and mentally) into top professional players [27]. In women’s football this is even more important because most women football players have a late start in playing football and do not have the same exposure to football at a young age that their men/boys counterparts have [28]. Consequently, as shown in other football codes, they may not develop appropriate movement patterns and movement quality, which may negatively impact on their football technique and quality as senior players [29]. This may also increase the risk of injury when playing [30]. Earlier exposure to sport (football included) would thus be expected to be associated with better quality movement, which in women’s football would translate to better quality football, better performances and winning more matches, i.e., continuity of the game and winning. As African women football teams continue to struggle against their counterparts from better resourced countries, an emphasis on youth development seems a sensible long-term plan to improve the fortunes of African women’s football teams. However, the world football governing body, FIFA, and the continental governing body, CAF, already have programs available for developing youth football players and early talent identification through the member associations (MAs) such as the FIFA Talent Development Scheme, the FIFA Women Development Programme, the FIFA Forward Development Programme, the CAF African Schools Championship, and FIFA Football for Schools [2, 4, 31]. That stakeholders in this study would want more of these likely implies that they may not be aware of their existence or scope. It is also possible that even with many youth development programs available at continental and global governing body levels, poor implementation and resource allocation for these programs at grassroots levels in their Member Associations may explain why these are still considered a top priority by most of our cohort. As such, addressing possible top-down problems in implementation of existing African youth football programs utilizing the principles of stakeholder theory remains crucial. Further, this finding may also suggest that the available youth development programs do not align well with the stakeholders’ priorities for youth (girls’) football development. Perhaps more consultation with stakeholders such as from this study may better define how the available youth development programs can better address their needs for youth football development in girls.

Findings from this study show that optimising physical conditioning (strength, speed, power, and endurance) was the second highly relevant research area. This concurs well with growing calls globally that women and girls train and compete under different conditions from men, hence the need to be prepared and trained as such [32]. As an intense multidirectional and intermittent field sport, football requires a high level of physical conditioning [33, 34]. Therefore, understanding the physical demands of the game is important in developing specific and progressive strength and conditioning programs for women football players [33]. Notably, research in African women football players shows that they would benefit from structured conditioning programs [35]. However, implementing these training models such as those that involve periodisation requires resources (human and otherwise), which may not always be available for women footballers [36, 37]. This is because, even in high income countries, participants in women’s football (e.g., players, player support personnel) often do so on a part-time or voluntary basis [38, 39]. It would be interesting to see how physical conditioning training is optimised among African women footballers considering the demands of time, balancing of gender-related roles, and limited resources.

Another priority topic identified was the need to understand female specific health issues (such as the menstrual cycle, pregnancy, and menopause) of women in football. This mirrors previous research in which stakeholders in African women’s football expressed interest in learning more about the menstrual cycle’s effect on women football players [40]. To date, evidence on the influence of hormone fluctuations during the menstrual cycle on athletic performance and musculoskeletal injury risk is uncertain [41, 42]. However, menstrual related symptoms such as heavy bleeding, abdominal cramps and mood swings can affect training and performance [43–45]. Furthermore, guidelines for football training during and after pregnancy still leave a lot to be desired [46], and minimal work has been done on perimenopause and the menopause in women’s sport [47]. It is, however, interesting to note that if given an opportunity, stakeholders would invest the hypothetical US$1 million in research on development of youth players, improvement of technical skills, and injury prevention over female specific health related factors. This is likely because of the level of the game in the region, where the emphasis might still be on developing the fundamentals of the game, which would presumably achieve greater gains for teams over areas that may be perceived as offering marginal gains such as menstrual cycle phase-based training. This also reflects fundamental differences in priorities in different cohorts of women football stakeholders, and the need for international funders to consider local contexts in women’s football research and development funding.

Evidence shows that excellent technical skills are an important determinant of football success [48] and stakeholders in this study ranked improving technical skills as one of the highly relevant areas for targeted research. Technical skills include on-the-ball-performance actions such as ball control, passes, dribbles and tackles among many others [49]. To date, studies done on women football players show that they have different and lower scores in most technical skills when compared to men and boys’ football players [28, 50]. This is likely because of the gendered environment in which females play, which curtails their technical development or even predisposes them to injury [51]. Notably, African girls’ first exposure to football is nearly two years after their boy counterparts, they generally have less opportunities to play football, and they have relatively poor physical conditioning [35, 52]. Additionally, women athletes tend to have substandard access to athlete support personnel such as coaches, who are often inexperienced when available [29]. Therefore, improvements in technical skills in African women’s football would require input from the broader African football ecosystem to mitigate the challenges that women players face in achieving this and cultivating environments that are conducive for early technical skills development.

Prevention of injuries was also considered an important aspect on which to conduct further research. Given the consequences of injury such as financial and psychological cost of injury management, player unavailability, and the scarcity of athlete support personnel to support injury management in most African women’s teams [8, 53], stakeholders would want to have more robust injury prevention strategies. Unsurprisingly, injuries are the most researched area in women’s football [5]. According to the TRIPP framework, an iteration of van Mechelen’s model, injury prevention strategies should be preceded by establishing the extent of the injuries (severity and incidence) through surveillance and establishing the aetiology/mechanisms of these injuries [54]. Injury prevention strategies can then be implemented and monitored first under ideal conditions then later in real world settings that consider the context. However, most injury prevention research in women’s football is stuck on or before step 1 (establishing the extent of the problem), with very little longitudinal injury surveillance research being conducted in women’s football globally. Although a few studies have been carried out the broad area of injuries and the menstrual cycle among women football players in countries in the COSAFA region [10–12], more than 75% of this work has been conducted in Europe and the USA [25]. Therefore, in order to conduct more research on injury prevention strategies in African women’s football, it is important that more longitudinal injury surveillance research be conducted on this cohort. To date, most countries in the COSAFA region except South Africa have not received attention in women’s football literature [1, 55–57] and tournaments like the COSAFA Women’s Championships provide an important opportunity to conduct such surveillance research.

Areas that were ranked lowest for relevance in targeted research in African women’s football were research on transgender athletes; anti-doping; equity, diversity, and inclusion (EDI); and developing para/disability football. Though important areas, this suggests that in the hierarchy of needs in African women’s football, these are considered lower priority. This contrasts with other work in the United States [58], and policies in Australia which prioritise research on transgender athletes [59] as well as FIFA, World Anti-doping Agency (WADA), and CAF policy prioritising anti-doping [2, 60, 61]. While we can speculate about the possible reasons for this low ranking, it seems anti-doping policy and programs in Africa may not get prioritisation due to other pressing issues higher up on the policy agenda such as building beneficial partnerships with sponsors and improving professionalism in competitions [2, 62]. Similarly, the subject of transgender athletes and athletes with variations of sexual development which falls in the broad category of LGBTQIA+ rights remains a contentious issue in Africa. While being intersex and asexual is not illegal, same sex relations are criminalised in 31 of the 54 African countries [63, 64]. In the COSAFA region, LGBTQ individuals have equal rights under the law in South Africa, whilst public expression of lesbian, gay, bisexual, trans and queer identities can be penalised in countries such as Malawi, Zambia and Zimbabwe [63]. The low priority ranking may thus reflect the participants’ socio-cultural and religious beliefs, as well as an awareness of legislation in their respective countries. Concerning para-football, it is likely that as this is an emergent research avenue, even in higher resourced countries, para-football research has received less attention [5] and African women’s football must also catch up.

### Study limitations

The study findings are not without limitations. Firstly, the questionnaire used in this study was adopted from earlier research in a different context and was not revalidated for this study. Therefore, this may have limited how it captured the intended constructs. Secondly, these findings were collected from a sample of stakeholders (23.5%) participating at two top tier competitions for women’s football in the Southern African region. This means the reported priorities may not reflect those of stakeholders in grassroots women’s football who are the majority. Furthermore, any generalisation to the rest of the African continent should be made with caution. Thirdly, because of the voluntary nature of the study, there is a possibility of recruitment selection bias. This means those willing to participate might have considerably differed in various characteristics from those who declined to participate in a study. Lastly, no inferential statistical comparisons between different stakeholder groups were carried out due to the extreme sample size imbalance. We only reported on descriptive statistics with the plan of carrying out robust comparisons between larger groups in the future.

## Conclusion

Overall, this study highlighted what stakeholders consider relevant research priority areas in women football in the COSAFA region. The most important area is development of youth players while health related issues such as optimising physical conditioning, understanding female related health issues and injury prevention were also in the top five. Anti-doping and transgender athletes or athletes with differences of sexual development ranked lowly as targeted research areas. As global funding agencies decide how to further research in women’s football, it is important that stakeholder perspectives be taken into consideration as they will help guide context relevant and optimal usage of resources, especially in countries and cultures where women’s football is less developed. Relatively fewer studies have been carried out in African women’s football on the identified priority areas; hence more work is warranted. 

## Supplementary Information

Supplementary Material 1.

Supplementary Material 2.

## Data Availability

De-identified data collected in the study will be made available following publication upon reasonable request to the corresponding author on [nonhlanhla.s.mkumbuzi@durham.ac.uk](mailto:nonhlanhla.s.mkumbuzi@durham.ac.uk%20).

## Abbreviations

CAF: Confederation of African Football
CAFWCL: Confederation of African Football Women’s Champions League
FIFA: International Federation of Association Football
COSAFA: Council of Southern Africa Football Associations
STROBE: Strengthening the Reporting of Observational Studies in Epidemiology
PT: Physiotherapists
S&C coach: Strength and Conditioning Coach
LGBTQIA+: Lesbian, Gay, Bisexual, Transgender, Queer, Intersex, Asexual and Plus
WADA: World Anti-doping Agency
TRIPP: Translating Research into Injury Prevention Practice
SAFA: South African Football Association
