# Supplementary material for: Setting the research agenda for Council of Southern Africa Football Associations (COSAFA) women’s football: stakeholder perspectives
Source: BMC Sports Sci Med Rehabil. 2026 Apr 17;18:254. doi: 10.1186/s13102-026-01692-y (PMC13220478; doi:10.1186/s13102-026-01692-y)
Supplement: Supplementary file 1 — Supplementary Material 1. [file 13102_2026_1692_MOESM1_ESM.docx]

**Supplementary Information 2 – Study questionnaire**

| **SECTION A: DEMOGRAPHIC AND TRAINING DATA** | | | | |
| --- | --- | --- | --- | --- |
| **What country are you from?** | | |  | |
| **How old are you?** |  | **What is your gender?** | | Male  Female  Other  Prefer not to say |
| **What is the highest level of education/training you have attained?** | Primary school  Secondary school  Tertiary- Diploma  Tertiary- Bachelors  Tertiary- Masters  Tertiary- Doctorate | | | |
| **What is your position in the football team?** | Player/athlete  Coach  Team/squad physiotherapist  Team/squad doctor  Team/squad welfare manager  Team/squad strength and conditioning coach  Match official (e.g. referee, umpire)  Administrator (e.g. Chef de Mission, country/sport representative)  Other (specify)………………………………………………………. | | | |
| **When did you start participating in football?** |  | | | |
| **At what level are you currently involved in football?** | Amateur  Semi-professional  Professional  Other (specify)……………………………………. | | | |
| **In which country is your current football club based?** |  | | | |

| **SECTION B** | | | | |
| --- | --- | --- | --- | --- |
| **Which of the following topic areas do you think would be relevant to do targeted research on African women football players?**  Please answer each question by placing an **(X)** in the appropriate column | | | | |
|  | Highly relevant | Relevant | Somewhat relevant | Not at all relevant |
| Improving technical skills |  |  |  |  |
| Enhancing tactical knowledge and application |  |  |  |  |
| Optimising physical conditioning (strength, speed, power, and endurance) |  |  |  |  |
| Anti-doping |  |  |  |  |
| Optimising diet and nutrition |  |  |  |  |
| Optimising recovery systems |  |  |  |  |
| Improving psychological attributes and skills |  |  |  |  |
| How to prevent injuries |  |  |  |  |
| How to come back stronger after injury |  |  |  |  |
| Developing youth players (technical, tactical, mental, physical) |  |  |  |  |
| Developing para/disability football |  |  |  |  |
| Understanding the impact of potential female health related factors (e.g. menstrual cycle, pregnancy, menopause) |  |  |  |  |
| Enhancing leadership and coaching behaviours |  |  |  |  |
| Developing equipment/technologies targeting high performance female athletes |  |  |  |  |
| Preparing for and transitioning into retirement (mental and physical health) |  |  |  |  |
| Transgender athletes and athletes with variations of sexual development |  |  |  |  |
| Investigating equity, diversity, and inclusion in football |  |  |  |  |

**Please add any area(s) not listed above that you consider highly relevant for targeted research.**

1. ………………………………………………………………………………………………………………
2. ………………………………………………………………………………………………………………
3. ………………………………………………………………………………………………………………
4. ………………………………………………………………………………………………………………

**If you were given USD $1 million to do research on African women’s football, what are the top three (3) research areas that you would invest in? (Choose any three (3) below)**

Improving technical skills

Enhancing tactical knowledge and application

Optimising physical conditioning (strength, speed, power, and endurance)

Anti-doping

Optimising diet and nutrition

Optimising recovery systems

Improving psychological attributes and skills

How to prevent injuries

How to come back stronger after injury

Developing youth players (technical, tactical, mental, physical)

Developing para-football

Understanding the impact of potential female health related factors (e.g. menstrual cycle, pregnancy, menopause)

Enhancing leadership and coaching behaviours

Developing equipment/technologies targeting high performance female athletes

Preparing for and transitioning into retirement (mental and physical health)

Transgender athletes and athletes with variations of sexual development

Investigating equity, diversity, and inclusion in football

Other (Specify)…………………………………………………………………………………………

Other (Specify)…………………………………………………………………………………………

Other (Specify)…………………………………………………………………………………………

**THANK YOU FOR PARTICIPATING IN THIS SURVEY ☺**
