# Supplementary material for: Setting the research agenda for Council of Southern Africa Football Associations (COSAFA) women’s football: stakeholder perspectives
Source: BMC Sports Sci Med Rehabil. 2026 Apr 17;18:254. doi: 10.1186/s13102-026-01692-y (PMC13220478; doi:10.1186/s13102-026-01692-y)
Supplement: Supplementary file 2 — Supplementary Material 2. [file 13102_2026_1692_MOESM2_ESM.docx]

**Supplementary Information 1:** STROBE Statement—checklist of items that should be included in reports of observational studies

|  | Item No. | Recommendation | Page  No. | Relevant text from manuscript |
| --- | --- | --- | --- | --- |
| **Title and abstract** | 1 | (*a*) Indicate the study’s design with a commonly used term in the title or the abstract | 2 | Study design described as cross-sectional |
|  |  | (*b*) Provide in the abstract an informative and balanced summary of what was done and what was found | 2 | 141 participants from 10 countries completed a questionnaire ranking research priorities. Top priorities: youth development (82%), physical conditioning (79%), female health (76%). Funding priorities: youth development (17%), technical skills (16%), injury prevention (8%). |
| Introduction | | | |  |
| Background/rationale | 2 | Explain the scientific background and rationale for the investigation being reported | 4-5 | To date, studies to identify stakeholder views on women’s sports have been carried out mostly in stakeholders from high income countries. Identifying stakeholders’ research priorities in African women football is important as this can inform research directions and funding priorities. |
| Objectives | 3 | State specific objectives, including any prespecified hypotheses | 5 | Identify specific areas which stakeholders of African women’s football in the Southern African region consider as priorities in football medicine and performance research. |
| Methods | | | |  |
| Study design | 4 | Present key elements of study design early in the paper | 6 | Descriptive, cross-sectional survey |
| Setting | 5 | Describe the setting, locations, and relevant dates, including periods of recruitment, exposure, follow-up, and data collection | 6 | Study population consisted of women football players and their support personnel participating in two competitions of the Council of Southern Africa Football Associations in 2022. |
| Participants | 6 | (*a*) *Cross-sectional study*—Give the eligibility criteria, and the sources and methods of selection of participants | 6 | Voluntary participation of women football players and their support personnel (above 18 years old) |
| Variables | 7 | Clearly define all outcomes, exposures, predictors, potential confounders, and effect modifiers. Give diagnostic criteria, if applicable | N/A | N/A |
| Data sources/ measurement | 8* | For each variable of interest, give sources of data and details of methods of assessment (measurement). Describe comparability of assessment methods if there is more than one group | N/A | N/A |
| Bias | 9 | Describe any efforts to address potential sources of bias | 17 | Participants were recruited from all 14 member associations of the Council of Southern Africa Football Associations to ensure a diverse and representative sample of players and support personnel |
| Study size | 10 | Explain how the study size was arrived at | 6 | Study size was determined by recruiting a diverse sample from multiple countries/teams, with participation scheduled conveniently and additional voluntary participation encouraged |

Continued on next page

| Quantitative variables | 11 | Explain how quantitative variables were handled in the analyses. If applicable, describe which groupings were chosen and why | 7 | Normally distributed data were reported as mean ± SD; non-normal data as median [IQR] |
| --- | --- | --- | --- | --- |
| Statistical methods | 12 | (*a*) Describe all statistical methods, including those used to control for confounding | N/A | N/A |
|  |  | (*b*) Describe any methods used to examine subgroups and interactions | N/A | N/A |
|  |  | (*c*) Explain how missing data were addressed | 9 | Reported as other |
|  |  | (*d*) *Cross-sectional study*—If applicable, describe analytical methods taking account of sampling strategy | N/A | N/A |
|  |  | (*e*) Describe any sensitivity analyses | N/A | N/A |
| Results | | | | |
| Participants | 13* | (a) Report numbers of individuals at each stage of study—eg numbers potentially eligible, examined for eligibility, confirmed eligible, included in the study, completing follow-up, and analysed | 8 | 141 participants |
|  |  | (b) Give reasons for non-participation at each stage | N/A | N/A |
|  |  | (c) Consider use of a flow diagram | N/A | N/A |
| Descriptive data | 14* | (a) Give characteristics of study participants (eg demographic, clinical, social) and information on exposures and potential confounders | 8 | Participants included football players (40%), coaches (12%), referees (4%), medical doctors (2%), physiotherapists (1%), team managers (3.5%), administrators (13.5%), and not specified (23%) |
|  |  | (b) Indicate number of participants with missing data for each variable of interest | N/A | N/A |
|  |  | (c) *Cohort study*—Summarise follow-up time (eg, average and total amount) | N/A | N/A |
| Outcome data | 15* | *Cross-sectional study—*Report numbers of outcome events or summary measures | 8 | 141 out of 350 participants (approximately 23.5) |
|  |  | (*a*) Give unadjusted estimates and, if applicable, confounder-adjusted estimates and their precision (eg, 95% confidence interval). Make clear which confounders were adjusted for and why they were included | N/A | N/A |
|  |  | (*b*) Report category boundaries when continuous variables were categorized | N/A | N/A |
| Main results | 16 | (*c*) If relevant, consider translating estimates of relative risk into absolute risk for a meaningful time period | N/A | N/A |
|  |  |  |  |  |
|  |  |  |  |  |

Continued on next page

| Other analyses | 17 | Report other analyses done—eg analyses of subgroups and interactions, and sensitivity analyses | N/A | N/A |
| --- | --- | --- | --- | --- |
| Discussion | | | | |
| Key results | 18 | Summarise key results with reference to study objectives | 2, 9-12 | Top research priorities among participants were youth development (82%), physical conditioning (79%), female health (76%), technical skills (75%), and injury recovery (75%). Funding preferences focused on youth development (17%), technical skills (16%), and injury prevention (8%) |
| Limitations | 19 | Discuss limitations of the study, taking into account sources of potential bias or imprecision. Discuss both direction and magnitude of any potential bias | 17 | Voluntary participation may have introduced selection bias in recruitment |
| Interpretation | 20 | Give a cautious overall interpretation of results considering objectives, limitations, multiplicity of analyses, results from similar studies, and other relevant evidence | 17 | Limitations include use of a questionnaire adapted from a different context without revalidation, and a sample limited to 23.5% of stakeholders from two top-tier competitions, which may not represent grassroots women’s football |
| Generalisability | 21 | Discuss the generalisability (external validity) of the study results | 17 | Any generalisation to the rest of the African continent should be made with caution. |
| Other information | |  | | |
| Funding | 22 | Give the source of funding and the role of the funders for the present study and, if applicable, for the original study on which the present article is based | 19 | Funded by the Member Associations and Women’s Football Divisions of the Fédération Internationale de Football Association (FIFA), Zurich, Switzerland. |

*Give information separately for cases and controls in case-control studies and, if applicable, for exposed and unexposed groups in cohort and cross-sectional studies.

**Note:** An Explanation and Elaboration article discusses each checklist item and gives methodological background and published examples of transparent reporting. The STROBE checklist is best used in conjunction with this article (freely available on the Web sites of PLoS Medicine at http://www.plosmedicine.org/, Annals of Internal Medicine at http://www.annals.org/, and Epidemiology at http://www.epidem.com/). Information on the STROBE Initiative is available at www.strobe-statement.org.
